# Malondialdehyde at the Crossroads of Oxidative Stress, Lipid Peroxidation, Ferroptosis, and Hematological Malignancies: A Narrative Review

**DOI:** 10.3390/biomedicines14081837

**Published:** 2026-08-15

**Authors:** Federica Li Pomi, Adele Bottaro, Giuseppe Murdaca, Fabio Stagno, Manlio Fazio, Sebastiano Gangemi, Alessandro Allegra

**Affiliations:** 1 Department of Precision Medicine in Medical, Surgical and Critical Care (Me.Pre.C.C.), University of Palermo, 90127 Palermo, Italy; federicalipomi@hotmail.it; 2 Division of Hematology, Department of Human Pathology in Adulthood and Childhood “Gaetano Barresi”, University of Messina, 98125 Messina, Italy; bottarp15@gmail.com (A.B.); fabio.stagno@unime.it (F.S.); manlio.fazio@polime.it (M.F.); aallegra@unime.it (A.A.); 3 Department of Internal Medicine, University of Genova, 16126 Genova, Italy; 4 Allergology and Clinical Immunology, San Bartolomeo Hospital, 19038 Sarzana, Italy; 5 Allergy and Clinical Immunology Unit, Department of Clinical and Experimental Medicine, University of Messina, 98125 Messina, Italy; gangemis@unime.it

**Keywords:** malondialdehyde, oxidative stress, lipid peroxidation, ferroptosis, reactive oxygen species, acute myeloid leukemia, chronic myeloid leukemia, myeloproliferative neoplasms, lymphoma, multiple myeloma

## Abstract

Oxidative stress is increasingly recognized as a key contributor to the biology of hematological malignancies. Excessive production of reactive oxygen species disrupts redox homeostasis, promotes genomic instability, alters cellular signaling pathways, and influences disease progression, therapeutic response, and resistance mechanisms. Among the downstream consequences of oxidative stress, lipid peroxidation represents a major source of cellular injury, generating reactive aldehydes capable of amplifying molecular damage. Malondialdehyde, a stable end product of lipid peroxidation, has emerged as one of the most widely investigated biomarkers of oxidative damage in hematologic cancers. This review summarizes current evidence regarding the role of malondialdehyde across major hematological malignancies, including acute and chronic myeloid leukemias, Philadelphia-negative myeloproliferative neoplasms, lymphomas, and multiple myeloma. Available evidence consistently demonstrates significantly elevated MDA levels across major hematological malignancies, including acute and chronic myeloid leukemias, Philadelphia-negative myeloproliferative neoplasms, lymphomas, and multiple myeloma. Increased MDA concentrations are frequently associated with disease activity, relapse, impaired antioxidant defenses, thrombotic complications, treatment resistance, and therapy-related toxicity. Furthermore, experimental studies indicate that MDA accumulation closely parallels ferroptosis induction and may serve as a pharmacodynamic marker of lipid peroxide-mediated cell death. Overall, the reviewed literature identifies MDA as one of the most consistent biomarkers of oxidative stress and lipid peroxidation in hematological cancers. Although methodological standardization remains necessary, MDA appears to have potential diagnostic, prognostic, and therapeutic relevance and may contribute to identifying redox vulnerabilities that can be exploited by emerging ferroptosis-based treatment strategies.

## 1. Introduction

Oxidative stress (OS) is a central biological process in cancer biology, which reflects the disruption of cellular redox homeostasis caused by an imbalance between the production of reactive oxygen species (ROS) and the capacity of antioxidant systems to neutralize them or repair the resulting molecular damage [1].

Beyond cancer biology, oxidative stress has emerged as a fundamental mechanism involved in immune regulation, aging, chronic inflammation, and the development of several age-related disorders. Recent evidence indicates that redox imbalance profoundly influences immunometabolic pathways by modulating immune-cell activation, mitochondrial function, cellular energy metabolism, and inflammatory signaling.

Persistent oxidative stress may contribute to cellular senescence, impaired tissue homeostasis, and dysregulated immune responses, thereby creating a biological environment that favors both age-related degeneration and tumor development.

These observations support the concept that oxidative stress represents a common pathogenic denominator linking inflammation, immune dysfunction, aging, and malignant transformation, further emphasizing the clinical relevance of biomarkers reflecting oxidative damage and lipid peroxidation [2,3].

Under physiological conditions, ROS act as signalling mediators involved in cell proliferation, differentiation, immune responses, and metabolic adaptation, whereas persistent or excessive ROS generation can damage DNA, proteins, lipids, and cellular membranes [4].

This dual biological role is particularly relevant in malignant cells, in which redox alterations may contribute both to tumor-promoting signalling and to the accumulation of oxidative damage that shapes disease evolution and therapeutic vulnerability [5].

Cancer cells frequently display increased metabolic activity, mitochondrial dysfunction, inflammatory signalling, oncogene-driven ROS generation, and altered antioxidant defenses, all of which may converge toward a sustained pro-oxidant state [6]. Therefore, OS is not only a consequence of malignant transformation, but also a potential contributor to genomic instability, microenvironmental remodeling, clonal selection, and resistance or sensitivity to anticancer therapies [7,8].

Among the different molecular targets of OS, lipids represent a particularly important substrate because polyunsaturated fatty acids (PUFAs) in cellular and organelle membranes are highly susceptible to free radical-mediated oxidation [9].

Lipid peroxidation (LPO) is a self-propagating chain reaction initiated by ROS-mediated hydrogen abstraction from PUFAs, followed by oxygen addition and formation of lipid peroxyl radicals and lipid hydroperoxides [10]. Once initiated, this process can compromise membrane architecture, modify membrane fluidity, interfere with receptor and transporter function, and generate secondary electrophilic products capable of diffusing beyond the initial site of oxidative injury [11].

These lipid-derived products may amplify oxidative damage by forming covalent adducts with proteins, nucleic acids, and phospholipids, thereby linking membrane oxidation to broader disturbances in cellular function and signal transduction [12]. For this reason, LPO represents one of the most biologically informative consequences of OS and has been increasingly investigated as both a marker and a mediator of redox imbalance in cancer [13].

Malondialdehyde (MDA) is one of the best-known end products of LPO and is generated mainly during the oxidative degradation of PUFAs [14]. Because of its relative stability compared with short-lived ROS, MDA has been widely used as an indirect biomarker of lipid oxidative damage in biological fluids and tissues [9].

MDA should not be considered merely a passive degradation product, since it can react with proteins and nucleic acids, thus contributing to the formation of adducts that may alter protein function, enzyme activity, membrane-associated processes, and genomic integrity [5].

In experimental and clinical research, MDA is often measured to evaluate the burden of LPO, although methodological issues must be considered because commonly used assays, such as thiobarbituric acid reactive substances-based methods, may lack absolute specificity for MDA [15]. In fact, MDA can be quantified using several analytical approaches. The most widely employed method is the thiobarbituric acid reactive substances (TBARS) assay, which is inexpensive and technically simple but may lack absolute specificity because other aldehydes and oxidation products can also react with thiobarbituric acid.

More accurate methodologies include high-performance liquid chromatography (HPLC), often coupled with fluorescence or ultraviolet detection, as well as gas chromatography–mass spectrometry (GC-MS) and liquid chromatography–mass spectrometry (LC-MS), which provide higher sensitivity and specificity. Differences in sample processing, biological matrices, analytical platforms, and calibration procedures may contribute to variability among studies and should be considered when comparing MDA concentrations across different clinical settings. Consequently, methodological standardization remains an important prerequisite for the broader clinical application of MDA as a biomarker of oxidative stress and lipid peroxidation.

In addition to methodological differences, several pre-analytical and clinical variables may contribute to the heterogeneity of MDA measurements reported across studies.

MDA concentrations may vary according to the biological matrix analyzed (serum, plasma, erythrocytes, peripheral blood cells, bone marrow samples, or tissue specimens), sample collection and storage procedures, and the timing of measurement relative to diagnosis or treatment.

Furthermore, patient-related factors such as age, sex, smoking status, dietary habits, inflammatory conditions, metabolic disorders, cardiovascular comorbidities, disease stage, tumor burden, and ongoing therapies may all influence systemic oxidative stress and consequently affect MDA levels. These sources of variability should be considered when comparing studies and may partly explain inconsistencies in the magnitude of MDA elevation reported in different hematological malignancies.

Several biomarkers have been proposed to assess oxidative stress and its biological consequences. Among these, lipid peroxidation can be evaluated through measurement of MDA, 4-hydroxynonenal (4-HNE), and F2-isoprostanes. Oxidative DNA damage is commonly assessed by 8-hydroxy-2′-deoxyguanosine (8-OHdG), whereas protein oxidation may be investigated through advanced oxidation protein products (AOPPs), protein carbonyls, and nitrotyrosine derivatives. Each biomarker provides complementary information regarding specific molecular targets of reactive oxygen species and the underlying mechanisms of oxidative injury.

Importantly, MDA should not be regarded exclusively as a lipid peroxidation product generated during oxidative stress. It may also arise during arachidonic acid metabolism associated with thromboxane A_2_ synthesis, particularly in conditions characterized by platelet activation and enhanced thrombotic activity. Consequently, elevated MDA concentrations may in some settings reflect not only oxidative membrane damage but also increased thromboxane generation and platelet-related biological processes.

Although several biomarkers display greater molecular specificity than MDA, including F2-isoprostanes for lipid peroxidation and 8-OHdG for oxidative DNA damage, MDA remains one of the most extensively investigated oxidative stress markers in both experimental and clinical studies. Its widespread use derives from its relative stability, the availability of standardized analytical methods, relatively low cost, and the large body of literature accumulated across different hematological malignancies.

Furthermore, the growing interest in ferroptosis has renewed attention toward MDA because it represents a downstream product of lipid peroxide accumulation and may therefore provide information that is directly relevant to ferroptosis-associated oxidative injury. For these reasons, despite its recognized limitations, MDA continues to represent a valuable biomarker for investigating the interplay between oxidative stress, lipid peroxidation, and hematological cancer biology.

The relevance of MDA in cancer derives from the fact that LPO products can influence several processes implicated in malignant transformation and tumor progression [1]. Oxidative damage to lipids and the accumulation of reactive aldehydes may affect cell signalling, membrane-dependent pathways, inflammatory responses, DNA integrity, and the balance between survival and regulated cell death. In this context, MDA has been investigated as a readout of oxidative damage in several oncologic settings and has often been interpreted as a surrogate marker of the intensity of LPO associated with cancer-related redox imbalance.

Hematologic malignancies provide a particularly relevant setting in which to examine OS and LPO because hematopoietic cells are highly dependent on tightly regulated redox homeostasis for self-renewal, differentiation, proliferation, and survival [16].

Disruption of redox balance within hematopoietic compartments may contribute to oxidative DNA damage, altered signalling, genomic instability, and functional impairment of normal and malignant hematopoietic cells [9]. In addition, the biology of hematologic tumors is closely connected to the bone marrow microenvironment, inflammatory networks, mitochondrial metabolism, and iron handling, all of which may influence ROS generation and lipid oxidative damage [17].

The measurement of MDA in this context is therefore biologically plausible, as it may reflect the downstream consequences of redox imbalance on lipid-rich cellular and subcellular membranes. Although available studies have explored MDA and other oxidative damage markers in hematologic cancer, the field still requires careful synthesis to clarify whether MDA is best interpreted as a marker of oxidative burden, disease biology, treatment-related injury, or vulnerability to specific redox-modulating strategies [9].

The increasing interest in ferroptosis has further reinforced the importance of LPO in hematologic cancer research [18]. Ferroptosis is an iron-dependent form of regulated cell death characterized by the accumulation of lipid peroxides and by failure of antioxidant systems that normally detoxify phospholipid hydroperoxides [12]. This process is mechanistically distinct from apoptosis and is strongly linked to iron metabolism, glutathione availability, glutathione peroxidase 4 activity, mitochondrial function, and lipid remodeling [18].

In hematologic malignancies, the ferroptosis framework is particularly attractive because malignant hematopoietic cells may show altered iron metabolism and redox adaptation, potentially creating a biological context in which LPO is both a marker of oxidative injury and a possible therapeutic liability [17]. Recent evidence has therefore emphasized the relevance of iron-dependent LPO and ferroptotic pathways as emerging mechanisms in leukemia and related hematologic neoplasms, supporting the need to better characterize LPO biomarkers within this disease area [19,20].

**Figure 1:**
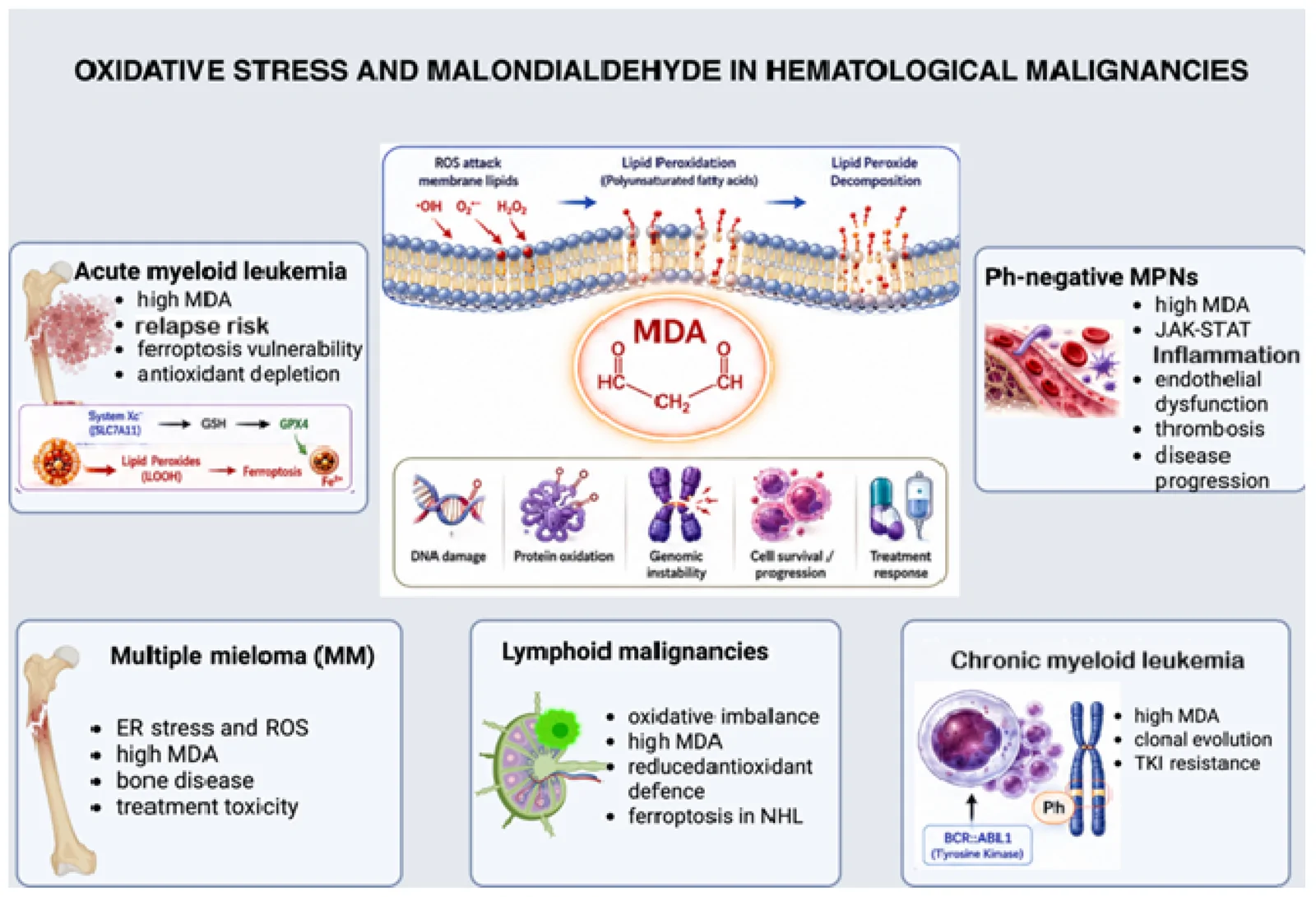
Biological relationship between oxidative stress, lipid peroxidation, malondialdehyde generation, and ferroptosis in hematological malignancies. Excessive ROS production promotes lipid peroxidation of PUFAs, leading to membrane damage and formation of reactive aldehydes, including MDA. MDA accumulation reflects oxidative injury and may contribute to protein and DNA modifications. Iron-dependent lipid peroxide accumulation further activates ferroptosis pathways through dysregulation of the SLC7A11–GSH–GPX4 axis. These interconnected processes contribute to disease progression, treatment resistance, and redox vulnerabilities in hematological malignancies.

Moving from these premises, MDA represents a rational focus for reviewing the interface between OS, LPO, and hematologic malignancies. Its biological relevance derives from its position downstream of ROS-mediated membrane lipid oxidation, its capacity to reflect cumulative oxidative damage, and its potential association with cellular dysfunction in malignant hematopoiesis [9] (Figure 1).

Accordingly, this paper focuses on MDA in hematologic malignancies with the aim of contextualizing current evidence, clarifying the biological rationale, and evaluating whether this LPO product may have value as a biomarker of OS, disease-associated redox imbalance, or therapeutic response in hematologic cancer research.

This narrative review was conducted through a structured search of the available literature on MDA, oxidative stress, lipid peroxidation, ferroptosis, and hematological malignancies. Searches were performed in PubMed, Scopus, and Web of Science databases up to June 2026.

The search strategy included combinations of the following keywords: “malondialdehyde”, “MDA”, “oxidative stress”, “lipid peroxidation”, “reactive oxygen species”, “ferroptosis”, “acute myeloid leukemia”, “chronic myeloid leukemia”, “myeloproliferative neoplasms”, “lymphoma”, and “multiple myeloma”.

Priority was given to original articles, translational studies, clinical investigations, and recent reviews published in English. Seminal studies considered essential for understanding the biological role of MDA were also included regardless of publication year. Additional references were identified through manual screening of bibliographies from relevant publications.

Despite the growing literature on oxidative stress, lipid peroxidation, and ferroptosis in hematological malignancies, these topics have generally been addressed separately, while the potential role of MDA as a common molecular link has received comparatively limited attention.

Previous reviews have mainly focused on the pathogenic role of ROS, antioxidant dysregulation, or ferroptosis-related pathways within specific hematologic diseases. In contrast, the present review provides an integrated overview of MDA across a broad spectrum of hematological malignancies, including leukemias, Philadelphia-negative myeloproliferative neoplasms, lymphomas, and multiple myeloma. By bringing together clinical, translational, and experimental evidence, we aim to highlight the value of MDA not only as a biomarker of lipid peroxidation and oxidative stress, but also as a potential indicator of disease activity, therapeutic response, treatment-related toxicity, and ferroptosis-associated redox vulnerabilities. This integrated perspective may facilitate a better understanding of the biological and clinical relevance of MDA and identify areas requiring further investigation before its implementation in routine clinical practice.

## 2. Oxidative Stress and Malondialdehyde in Hematologic Malignancies

### 2.1. Acute Myeloid Leukemia

Acute myeloid leukemia (AML) is an aggressive hematologic malignancy characterized by the clonal expansion of immature myeloid progenitors and suppression of normal hematopoiesis. A growing body of research suggests that OS plays a significant role in the pathophysiology of AML by fostering metabolic rewiring, leukemic cell survival, genomic instability, treatment resistance, and disease recurrence.

Leukemic development is influenced by oxidative damage caused by ROS produced by mitochondria, NADPH oxidases, and oncogenic signaling pathways [9]. Among the various oxidative injury biomarkers, MDA has become one of the most well-studied markers of OS in AML and may offer insights into the biology of the disease, prognosis, risk of relapse, and responsiveness to treatment [21].

#### 2.1.1. Oxidative Stress and Lipid Peroxidation in AML

AML cells exhibit increased ROS production compared with normal hematopoietic progenitors due to dysregulated mitochondrial metabolism, aberrant signaling pathways, and altered antioxidant systems [22].

Although moderate ROS concentrations may support leukemic proliferation and survival, excessive OS induces damage to cellular macromolecules and contributes to disease treatment [23].

Rasool et al. provided clinical evidence for the role of LPO in AML by showing significantly lower levels of antioxidant enzymes such as catalase, reduced glutathione (GSH), glutathione peroxidase (GPx), and superoxide dismutase (SOD) in AML patients compared to healthy controls [24]. MDA concentrations in AML patients were around seven times greater than in controls in that study, suggesting severe systemic OS.

Leukemic cells may reside in a continuously oxidizing milieu with persistent ROS production and insufficient antioxidant compensation, according to the simultaneous loss of enzymatic and non-enzymatic antioxidant defenses [24]. These results are in line with previous findings that demonstrate OS causes leukemogenesis, mutagenesis, and DNA damage by continuously exposing hematopoietic cells to reactive oxygen intermediates [25].

#### 2.1.2. MDA and Disease Progression

A particularly important contribution to understanding the clinical significance of OS in AML derives from the work of Zhou et al. In a relatively large clinical cohort study including 102 AML patients evaluated both at diagnosis and relapse, they investigated oxidative stress biomarkers and documented persistently elevated MDA levels throughout disease evolution [21]. The authors demonstrated that plasma MDA concentrations were significantly higher in relapsed AML than in healthy subjects and remained elevated throughout disease evolution, suggesting a close relationship between LPO and leukemic progression.

Additionally, elevated levels of 8-hydroxydeoxyguanosine (8-OHdG) and advanced oxidation protein products (AOPP) were seen in relapsed patients, suggesting concomitant oxidative damage to DNA and proteins. Crucially, relapse markedly decreased total antioxidant capacity, indicating the gradual depletion of protective antioxidant systems during the course of the disease [21]. Thioredoxin (TRX), a key regulator of cellular redox homeostasis, was shown to be upregulated in the same study, which also showed that lower antioxidant capability and higher TRX expression were independently linked to shorter relapse-free survival [21].

These observations strongly suggest that OS is not merely a consequence of leukemic proliferation but may actively contribute to disease persistence, progression, and recurrence.

#### 2.1.3. MDA, Ferroptosis, and Redox Vulnerabilities in AML

As reported above, ferroptosis is a regulated form of cell death characterized by iron-dependent accumulation of lipid peroxides and progressive membrane damage. Recent studies have expanded current understanding of OS in AML by identifying ferroptosis as a therapeutically exploitable vulnerability [26].

MDA buildup is thought to be a sign of ferroptotic cell death since it is produced during LPO. The primary intracellular defensive mechanism against ferroptosis is the glutathione peroxidase 4 (GPX4)-glutathione (GSH)-SLC7A11 axis, which detoxifies lipid hydroperoxides before they transform into very reactive aldehydes like MDA [27].

There is mounting evidence that GPX4 activity is critical for the survival of AML cells, and that interruption of this pathway results in significant increases in MDA and other LPO products [28,29].

By altering the MAGEA6–AMPK–SLC7A11–GPX4 signaling cascade, hypomethylating drugs have been shown to work in concert with ferroptosis inducers to accelerate LPO and leukemic cell death [30].

In mechanistic in vitro studies using AML cell lines, Akiyama et al. demonstrated that GPX4 inhibition induces ferroptosis characterized by enhanced lipid peroxidation and MDA accumulation, and significant antileukemic effects, thereby increasing the susceptibility of leukemic cells to ferroptosis-based therapeutic strategies [31].

Studies investigating daunorubicin resistance have further demonstrated that resistant AML cells exhibit altered redox profiles and reduced LPO, whereas restoration of oxidative damage is accompanied by increased MDA production and renewed sensitivity to treatment [32]. These observations suggest that suppression of LPO may represent a survival mechanism in AML, whereas induction of MDA-generating oxidative damage may enhance therapeutic efficacy.

Several recent experimental studies have used MDA as a direct biomarker to monitor ferroptosis induction in AML. Using cytarabine-resistant AML cell models, Kong et al. demonstrated that restoration of ferroptotic sensitivity increased intracellular MDA levels together with iron accumulation and GPX4 suppression [33].

The study further identified the SIRT1–HMGB1–ACSL4 signaling pathway as a regulator of ferroptosis susceptibility and drug resistance. Likewise, nanoparticle-based therapeutic approaches designed to induce ferroptosis in AML cells significantly increased MDA concentrations together with Fe^2+^ accumulation and suppression of SLC7A11 and GPX4 activity [21].

More recently, Jian et al. demonstrated that treatment with the NEDD8-activating enzyme inhibitor MLN4924 (pevonedistat) induced ferroptosis in AML cells through inhibition of the SLC7A11/GPX4 axis [34]. MLN4924 treatment caused significant increases in ROS, intracellular Fe^2+^, and MDA together with depletion of cellular GSH pools. Significantly, leukemic cells were saved from treatment-induced death by the ferroptosis inhibitor ferrostatin-1, which reversed MDA accumulation and directly demonstrated that elevated MDA is a sign of ferroptotic lipid damage [3]. The usefulness of MDA as a pharmacodynamic biomarker for tracking ferroptosis-targeted treatments in AML is supported by these results.

In summary, the information that is now available consistently shows that AML is linked to severe OS, which is marked by increased oxidative damage to DNA and proteins, decreased antioxidant defenses, and higher LPO. MDA is markedly elevated in AML patients and seems to be associated with oxidative damage, relapse risk, and disease activity. LPO is linked to new therapeutic approaches that target redox vulnerabilities in AML, and recent mechanistic investigations have further validated MDA as a key indicator of ferroptosis.

Clinical studies consistently support an association between elevated MDA levels and disease activity, relapse, and oxidative stress burden in AML patients. In contrast, evidence linking MDA to ferroptosis-targeted therapeutic strategies is currently derived largely from preclinical studies. Therefore, while MDA appears promising as a biomarker of redox imbalance, its clinical utility for therapeutic monitoring requires further prospective validation (Table 1).

However, most available studies have evaluated relatively limited patient cohorts and employed different methodologies for MDA determination, making it difficult to establish universally applicable threshold values or fully define its independent prognostic significance.

### 2.2. Chronic Myeloid Leukemia

Chronic myeloid leukemia (CML) is a myeloproliferative neoplasm characterized by the presence of the Philadelphia chromosome and the expression of the constitutively active BCR::ABL1 tyrosine kinase, which represents the key molecular event responsible for leukemic transformation. BCR::ABL1 activity not only promotes cellular proliferation and clonal survival but also profoundly disrupts redox homeostasis through increased generation of ROS [50].

Excessive ROS production contributes to promoting disease progression and the emergence of tyrosine kinase inhibitor (TKI)-resistant clones. Among the various biomarkers of OS, MDA is one of the most extensively investigated indicators in CML [35].

Several clinical studies have documented significantly elevated MDA levels in patients with CML compared with healthy controls. In clinical studies involving CML patients, Ahmad et al. demonstrated that increased MDA concentrations are associated with impaired antioxidant defenses and markers of disease progression, including reduced glutathione, glutathione peroxidase, superoxide dismutase, and vitamin C, indicating substantial disruption of systemic redox balance [36]. Moreover, patients resistant to imatinib exhibited significantly higher MDA levels than treatment responders, suggesting a potential prognostic role for this biomarker in the assessment of therapeutic outcomes [37].

The association between MDA and disease progression has been further supported by studies reporting higher circulating levels of both MDA and nitrite in patients receiving second-generation TKIs, a population characterized by greater biological aggressiveness, more frequent imatinib resistance, and more advanced disease stages [51]. These findings support the hypothesis that oxidative stress contributes to the acquisition of additional genetic abnormalities and drives clonal evolution in CML.

Another important aspect concerns the direct role of BCR::ABL1 in ROS generation. The BCR::ABL1 oncoprotein stimulates multiple intracellular sources of free radicals, including mitochondrial and cytoplasmic NADPH oxidases, resulting in a persistent pro-oxidative state. Elevated MDA levels represent a direct consequence of ROS-induced LPO and reflect the extent of oxidative injury in leukemic cells [52].

Nevertheless, a number of experimental models have further shown that oxidative stress induction may be used therapeutically in CML. According to Kumar et al., administering α-tocopheryl succinate and all-trans retinoic acid to primary leukemic cells resulted in increased LPO, as demonstrated by elevated MDA levels, along with increased ROS generation, mitochondrial membrane depolarization, caspase activation, and DNA fragmentation. These results imply that leukemic cells may undergo apoptosis when LPO is regulated [53].

The significance of the ROS–MDA axis in CML biology has been further emphasized by recent research. In K562 cells, pharmacological manipulation of NADPH oxidase activity lowered MDA levels while restoring antioxidant enzyme activities, including superoxide dismutase, catalase, and glutathione peroxidase.

These changes were accompanied by increased apoptosis and decreased cell viability, confirming a functional link between OS, LPO, and leukemic cell survival [54].

Also in this disease, ferroptosis has emerged as a potential mechanism regulating CML cell survival. Experimental in vitro models in TKI-resistant CML models have shown that increased ferroptosis susceptibility is associated with GPX4 degradation and impairment of glutathione-dependent antioxidant defenses, thereby promoting LPO and oxidative damage [55]. Since MDA is a major end-product of LPO, its accumulation may serve as an indicator of ferroptosis-associated oxidative injury and ferroptotic cell death activation.

Overall, MDA appears particularly relevant in CML because of its association with BCR::ABL1-driven oxidative damage, disease progression, and resistance to tyrosine kinase inhibitors.

Assessment of MDA, in combination with other redox biomarkers, may therefore provide valuable information for disease monitoring and support the development of therapeutic strategies aimed at modulating OS pathways.

### 2.3. Philadelphia-Negative Myeloproliferative Neoplasms

Philadelphia-negative myeloproliferative neoplasms (MPNs), including polycythemia vera (PV), essential thrombocythemia (ET), and primary myelofibrosis (PMF), are clonal hematopoietic disorders characterized by chronic inflammation, aberrant cytokine production, constitutive activation of JAK-STAT signaling, and a markedly increased risk of thrombotic complications [56].

Increasing evidence suggests that oxidative stress contributes to the inflammatory and thrombotic phenotype of Philadelphia-negative MPNs and may participate in disease progression. Also in this case, among the various biomarkers of oxidative damage, MDA, has emerged as one of the most extensively investigated indicators of redox imbalance in MPNs [57].

As reported above, persistent ROS generation promotes oxidative damage thereby establishing a pro-inflammatory microenvironment that supports clonal evolution and disease progression [58]. Inflammation and OS are now considered key biological links between chronic inflammation, endothelial dysfunction, thrombosis, and malignant transformation in Ph-negative MPNs [59].

#### 2.3.1. Oxidative Stress and Lipid Peroxidation in Polycythemia Vera

Numerous studies have shown that OS is higher in PV sufferers than in healthy people. According to a study, PV patients had higher erythrocyte MDA concentrations. The authors hypothesized that elevated MDA reflected oxidative damage to the lipids in the erythrocyte membrane, which led to increased erythrocyte stiffness and decreased cellular deformability [38].

Because decreased erythrocyte flexibility can lead to microvascular dysfunction and thrombotic problems, which are characteristics of PV, the rheological effects of LPO may be clinically significant [60]. In rather small observational cohorts of patients with PV and ET, Durmus et al. demonstrated significantly elevated MDA concentrations compared with healthy controls and a reduction after cytoreductive treatment. They also demonstrated that newly diagnosed PV patients had significantly higher serum concentrations of total oxidative status (TOS), and oxidative stress index (OSI).

Notably, MDA levels were approximately six-fold higher than those measured in controls, confirming the presence of substantial systemic LPO [60]. After six months of treatment with phlebotomy, low-dose aspirin, and hydroxyurea when indicated, serum MDA, TOS, and OSI significantly decreased, suggesting that OS is closely linked to disease activity. Additionally, patients with a history of vascular events exhibited significantly higher OS indices than patients without thrombosis, supporting a potential relationship between redox imbalance and vascular complications [60].

#### 2.3.2. Oxidative Stress in Essential Thrombocythemia

The presence of significant OS in ET is also supported by strong data. When compared to healthy controls, Durmus et al.’s evaluation of OS in ET patients revealed lower total antioxidant status (TAS) and significantly higher serum levels of TOS, OSI, and MDA [39]. The work demonstrated a persistent imbalance between oxidant production and antioxidant capacity by showing that LPO is accompanied by the depletion of antioxidant defenses.

Crucially, cytoreductive therapy dramatically decreased MDA and OSI levels, indicating that OS is more closely linked to the underlying myeloproliferative disease than to related comorbidities [39]. Further linking oxidative damage to the pathophysiology of vascular problems, patients with prior vascular events showed higher OS measures than those without thrombosis.

These findings support the hypothesis that inflammation and enhanced LPO contribute to endothelial dysfunction, platelet activation, and thrombotic risk in ET [61].

A contribution to the field was provided by Musolino et al., who investigated OS biomarkers in patients with PV and ET. The authors demonstrated significantly increased levels of advanced oxidation protein products (AOPPs) and S-nitrosylated proteins in both disorders, providing direct evidence of oxidative and nitrosative stress [40].

Furthermore, ET patients with a history of thrombosis displayed significantly higher concentrations of advanced glycation end products (AGEs) than patients without thrombotic events. The authors proposed that OS may contribute directly to thrombogenesis through endothelial damage, chronic inflammation, vascular dysfunction, and platelet activation. This work provided some of the first evidence linking OS biomarkers with thrombotic risk in Philadelphia-negative MPNs and highlighted the potential relevance of redox dysregulation in disease pathophysiology [40].

#### 2.3.3. Oxidative Stress and Primary Myelofibrosis

Among Ph-negative MPNs, PMF appears to exhibit particularly profound oxidative abnormalities. Vener et al. demonstrated increased OS and reduced antioxidant capacity in patients with both primary and post-polycythemia myelofibrosis. Increased OS was associated with more advanced disease and higher levels of circulating homocysteine [42].

In a more recent study, Mangione et al. conducted a focused metabolomic investigation of PMF patients and found significant evidence of oxidative and nitrosative stress along with a dramatic depletion of important circulating antioxidants [41]. When compared to healthy controls, PMF patients showed a four-fold decrease in reduced glutathione and a 37-fold decrease in blood ascorbic acid. Concurrently, circulating MDA concentrations were almost five times greater than those found in controls, and the rise in MDA was accompanied by increased quantities of nitrite and nitrate, indicating persistent oxidative and nitrosative stress.

Significant changes in purine and pyrimidine metabolism, including increased levels of hypoxanthine, xanthine, uric acid, uridine, and creatinine, were shown in the same study [37], indicating concurrent mitochondrial dysfunction and metabolic impairment.

These observations support a model in which persistent OS contributes to genomic instability, bone marrow remodeling, fibrosis, and progression toward advanced disease stages.

In conclusion, inflammation and OS appear to establish a self-reinforcing cycle in which activated leukocytes, platelets, endothelial cells, and neoplastic clones continuously generate ROS and pro-inflammatory mediators [62]. This model is supported by the observation that OS markers remain elevated throughout disease evolution and may correlate with thrombotic risk, disease burden, and fibrosis progression [63].

Collectively, these findings suggest that MDA may serve as a clinically relevant biomarker for monitoring OS, evaluating disease burden, identifying patients at increased thrombotic risk, and potentially assessing response to therapeutic interventions targeting redox pathways in Ph-negative MPNs.

Nevertheless, the majority of studies were observational in nature and frequently assessed MDA together with other oxidative stress markers, making it difficult to determine whether MDA provides independent information beyond global measures of redox imbalance.

### 2.4. Hodgkin and Non-Hodgkin Lymphomas

Lymphomas comprise a heterogeneous group of lymphoid malignancies traditionally classified into Hodgkin lymphoma (HL) and non-Hodgkin lymphomas (NHL) [64]. Increasing evidence indicates that OS plays a pivotal role in lymphoma biology [65]. Among the various biomarkers of oxidative injury, MDA is an indicator of OS in both HL and NHL. Elevated MDA levels have been documented in newly diagnosed patients, during disease progression, and in long-term survivors, supporting the concept that redox dysregulation is a fundamental component of lymphoma pathogenesis [66,67].

#### 2.4.1. Hodgkin Lymphoma

Numerous investigations have shown that patients with HL experience severe OS. Reed–Sternberg cells, activated immune cells, the inflammatory tumor microenvironment, and increased mitochondrial dysfunction may all contribute to increased ROS generation [68].

Morabito and colleagues conducted one of the most detailed analyses of OS in HL. In a clinical case–control study of patients with newly diagnosed Hodgkin lymphoma, they reported significantly increased serum MDA concentrations together with reduced antioxidant capacity [43].

A severe systemic oxidant–antioxidant imbalance was confirmed in the same study by HL patients’ markedly elevated ROS levels and decreased total antioxidant capacity (TAC). It is interesting to note that MDA concentrations were marginally greater in HL than in NHL, indicating that LPO might be especially noticeable in Hodgkin lymphoma.

More recently, Marini et al. demonstrated increased OS in peripheral blood mononuclear cells from HL patients, characterized by enhanced mitochondrial ROS generation, altered endoplasmic reticulum homeostasis, and increased oxidative damage [44]. The authors also identified dysregulation of hexose-6-phosphate dehydrogenase (H6PD) and glucose-6-phosphate dehydrogenase (G6PD), supporting the existence of profound metabolic remodeling associated with OS in HL.

Oxidative damage may persist long after successful therapy. Williams et al. demonstrated that long-term survivors of childhood Hodgkin lymphoma exhibit increased circulating markers of OS and inflammation decades after diagnosis. Elevated MDA and inflammatory biomarkers were associated with impaired neurocognitive performance, suggesting that persistent oxidative injury may contribute to long-term neurological sequelae in HL survivors [69].

These observations support the hypothesis that persistent LPO products such as MDA may contribute to the long-term complications observed in HL survivors. Thus, elevated MDA levels reflect disease-associated OS in HL and could potentially serve as biomarkers of biological activity and treatment-related sequelae.

#### 2.4.2. Non-Hodgkin Lymphomas

OS has also been extensively implicated in the pathogenesis of NHL, particularly in aggressive B-cell lymphomas. Increased ROS production may promote activation of oncogenic pathways, immune escape, and resistance to apoptosis [65].

OS may be a factor in the aggressiveness of disease, according to a number of studies. In a study of patients with recently diagnosed aggressive B-cell non-Hodgkin lymphoma, Haddouche et al. found markedly elevated levels of protein carbonyl and MDA, which is consistent with increased oxidative damage [45].

Additionally, patients showed reduced plasma TAC, which suggests that the systemic antioxidant defense network is compromised. Increased levels of oxidized LDL derivatives and nitric oxide generation coincided with the rise in MDA, indicating a widespread oxidative and inflammatory dysregulation in aggressive NHL. According to the researchers, high OS may cause lymphomagenesis by damaging DNA, encouraging mutagenesis, and triggering inflammatory pathways [45].

Additional evidence was provided by Zahzeh et al., who investigated patients with aggressive non-Hodgkin lymphoma exposed to pesticides and found significantly increased MDA levels together with reduced oxygen radical absorbance capacity (ORAC), glutathione peroxidase activity, catalase activity, and other antioxidant defenses, indicating pronounced oxidative imbalance associated with pesticide exposure [46].

Also in this case, recent studies have highlighted the importance of ferroptosis in NHL [70,71]. Chen et al. demonstrated that combined treatment with artesunate and sorafenib induced marked ferroptosis in NHL cell lines, accompanied by increased LPO, elevated MDA levels, increased ROS accumulation, and depletion of intracellular glutathione.

Furthermore, pharmacological induction of ferroptosis enhanced lymphoma cell death, whereas ferroptosis inhibition reduced apoptosis, suggesting functional crosstalk between LPO and programmed cell death pathways. The same study identified STAT3 as an important regulator of ferroptosis in NHL cells. These findings suggest that MDA may serve not only as a marker of oxidative damage but also as a pharmacodynamic biomarker for therapies targeting ferroptosis in aggressive NHL [72].

In lymphoma, MDA appears to reflect the interplay between tumor-associated inflammation, microenvironmental remodeling, and oxidative damage, while emerging data suggest a potential link with ferroptosis-related therapeutic approaches.

Increased MDA levels have been consistently documented in newly diagnosed patients and are frequently associated with reduced antioxidant defenses, increased ROS generation, and disease-related metabolic alterations.

Collectively, these observations support the use of MDA as a clinically relevant biomarker of OS in lymphoid malignancies and suggest that targeting redox pathways may represent a promising therapeutic approach in both Hodgkin and non-Hodgkin lymphomas [73].

Notably, the relationship between MDA and ferroptosis in lymphoma is currently supported predominantly by experimental evidence. Clinical studies have mainly documented increased MDA levels as indicators of oxidative stress, whereas the prognostic and therapeutic implications of these observations remain insufficiently characterized.

### 2.5. Multiple Myeloma

Multiple myeloma (MM) is a clonal plasma cell malignancy characterized by uncontrolled expansion of neoplastic plasma cells within the bone marrow microenvironment, leading to anemia, osteolytic bone disease, renal impairment, immunodeficiency, and eventual organ dysfunction [74].

Increasing evidence indicates that OS is deeply involved in myeloma biology, influencing genomic instability, plasma cell survival, interactions with the bone marrow niche, angiogenesis, disease progression, and resistance to therapy [75]. ROS are generated both intrinsically by metabolically active myeloma cells and extrinsically through inflammatory and stromal cell interactions, creating a redox imbalance that favors tumor growth. MDA has emerged as a marker in MM and may reflect disease burden, oxidative injury, treatment-related toxicity, and disease-associated complications [47,48].

#### 2.5.1. Oxidative Stress and Malondialdehyde in Multiple Myeloma

The malignant plasma cell clone is characterized by intense immunoglobulin synthesis that imposes substantial endoplasmic reticulum stress and promotes excessive production of ROS, contributing to disruption of cellular redox homeostasis and activation of oncogenic signaling pathways [76].

One of the most reproducible observations in MM patients is the increase in MDA [77]. In one of the earliest investigations, Zima et al. documented significantly elevated LPO together with reduced antioxidant enzyme activity in patients with MM, suggesting a persistent oxidant–antioxidant imbalance associated with disease pathophysiology [78].

Sharma et al. later showed that MM patients had markedly elevated serum MDA levels together with dereased levels of both enzymatic and non-enzymatic antioxidants, such as SOD, GPx, catalase, vitamins C, and vitamin E [79]. Mehdi et al. published similar results, showing reduced Cu/Zn-SOD activity and raised blood MDA levels in MM patients prior to therapy, suggesting impairment of antioxidant defense systems because of increased OS [80]. The occurrence of a systemic pro-oxidative state in MM was further supported by Lodh et al., who showed markedly elevated serum MDA concentrations linked to inflammatory activation and decreased antioxidant enzyme activity [81].

Smirnova et al. demonstrated that erythrocyte MDA concentrations were significantly increased in patients with stage II and stage III MM and that MDA levels rose progressively with advancing disease stage, suggesting a relationship between LPO and tumor burden [47].

The same study showed that increased MDA levels were accompanied by inadequate antioxidant compensation, indicating that OS intensifies during disease progression. Interestingly, plasma MDA concentrations remained stable in some patients despite significant oxidative damage at the cellular level, suggesting that circulating antioxidant systems may partially buffer systemic LPO products [47].

Additional evidence was provided by Khadem-Ansari et al., who evaluated newly diagnosed stage I MM patients and reported significantly higher serum MDA concentrations than in healthy controls, confirming that OS occurs even during early phases of disease evolution [48]. In that cohort, elevated MDA levels were associated with increased serum copper and ceruloplasmin concentrations together with decreased zinc levels and reduced Cu/Zn-SOD activity, further supporting the concept that MM is characterized by disturbed redox regulation. Notably, the authors proposed that OS markers could contribute to monitoring disease activity because alterations were already detectable in stage I disease before overt progression occurred [48].

Finally, Gangemi et al. showed that patients with plasma cell disorders and overt MM exhibited increased levels of OS biomarkers, including MDA, compared with healthy controls, and that these abnormalities were particularly pronounced in patients with bone disease [49].

These findings are biologically relevant because OS contributes directly to osteoclast activation, bone destruction, and remodeling abnormalities that characterize myeloma-associated skeletal disease [82]. The same study showed concomitant reductions in GSH-Px, catalase, and SOD levels together with increased nitric oxide concentrations, confirming a generalized shift toward oxidation. Multivariate analysis identified elevated MDA as an independent variable associated with MM diagnosis, while receiver operating characteristic analysis revealed an area under the curve of 0.967 for MDA, suggesting excellent discriminative potential.

These findings suggest that MDA may provide clinically relevant information regarding disease burden, skeletal involvement, and treatment-associated oxidative toxicity in MM.

#### 2.5.2. MDA and Treatment-Related Complications

Complications that arise with MM treatment may also be caused by oxidative damage. When compared to healthy controls, Dos Santos et al.’s evaluation of MM patients receiving autologous hematopoietic stem cell transplantation revealed a persistent hyperoxidative state during the transplantation process, which was made worse by the conditioning protocol [83]. Conditioning chemotherapy decreased antioxidant enzyme activity while increasing MDA concentrations, indicating that oxidative damage plays a role in transplant-related toxicity. Interestingly, engraftment kinetics and OS parameters measured prior to transplantation correlated, suggesting that redox biomarkers may have predictive value in the context of transplantation [83].

Proteasome inhibitors may also induce oxidative damage. Evidence derived from animal and preclinical models of carfilzomib exposure demonstrated increased tissue MDA concentrations associated with oxidative cardiotoxicity. Authors demonstrated that increases in tissue MDA concentrations were accompanied by depletion of GSH and catalase activity, supporting the contribution of LPO to treatment-associated cardiotoxicity [84].

Although derived from preclinical models, these observations are consistent with the clinical evidence linking proteasome inhibition to OS-mediated tissue injury.

In conclusion, elevated MDA concentrations have been documented across different disease stages, in newly diagnosed patients, during autologous transplantation, and in treatment-related complications. The progressive increase in MDA with advancing disease burden and its association with reduced antioxidant capacity suggest that LPO is not merely an epiphenomenon but may actively participate in myeloma pathogenesis and progression [85].

Consequently, MDA represents a promising biomarker for assessing OS in MM and may provide clinically relevant information regarding disease activity, therapeutic toxicity, and complication risk (Table 2).

## 3. Conclusions

OS is increasingly recognized as a biologically relevant feature of hematologic malignancies, where it may contribute to altered hematopoietic signaling, metabolic adaptation, disease progression, and treatment response. Within this redox framework, LPO represents one of the most important downstream consequences of excessive ROS generation, because it directly affects membrane integrity and generates reactive aldehydes capable of amplifying oxidative injury. Among these products, MDA has emerged as one of the most extensively investigated biomarkers of LPO in hematologic cancer research.

The available evidence indicates that MDA is frequently increased across different hematologic malignancies and is often accompanied by reduced antioxidant defenses, enhanced ROS production, protein oxidation, nitrosative stress, inflammatory activation, and disease-related metabolic alterations.

These findings support the concept that MDA may reflect a broader disruption of redox homeostasis rather than an isolated biochemical abnormality. In several disease contexts, increased MDA levels have been associated with disease activity, progression, therapeutic resistance, treatment-related toxicity, thrombotic complications, or long-term sequelae. Therefore, MDA may provide useful information on the oxidative burden associated with malignant hematopoiesis [86].

However, MDA should not be interpreted as a stand-alone diagnostic or prognostic marker. Furthermore, the clinical interpretation of MDA levels should take into account several biological and environmental factors that may influence systemic oxidative stress independently of hematological malignancies. Age-related changes in redox homeostasis, sex-associated differences in antioxidant defenses, dietary habits, smoking status, alcohol consumption, obesity, diabetes mellitus, cardiovascular disease, chronic inflammatory conditions, and other comorbidities may all contribute to variability in circulating MDA concentrations.

Consequently, elevated MDA levels should be interpreted within the broader clinical context and, whenever possible, alongside other oxidative stress biomarkers and disease-specific parameters. Future studies should incorporate appropriate adjustment for these potential confounding factors to better define the independent diagnostic and prognostic value of MDA in hematological malignancies.

MDA levels may be influenced by disease subtype, tumor burden, inflammatory status, iron metabolism, comorbidities, treatment exposure, sample type, and analytical methodology. In addition, commonly used assays for MDA assessment, particularly thiobarbituric acid reactive substances-based methods, may lack absolute specificity. For these reasons, the clinical interpretation of MDA is likely to be more informative when integrated with other markers of oxidative damage, antioxidant capacity, inflammatory activation, iron handling, and disease-specific molecular features [87,88].

The growing interest in ferroptosis has further strengthened the relevance of LPO and MDA in hematologic malignancies. Since ferroptosis is driven by iron-dependent accumulation of lipid peroxides and failure of antioxidant detoxification systems, MDA may serve as a useful pharmacodynamic marker of ferroptosis-associated lipid damage in experimental and translational settings. This is particularly relevant in hematologic tumors characterized by altered redox adaptation, increased oxidative burden, or disturbed iron metabolism [18,89]. Nevertheless, the precise role of MDA in ferroptosis-based therapeutic strategies remains to be fully clarified.

Despite the overall consistency of findings showing increased MDA levels across hematological malignancies, several limitations of the available evidence should be acknowledged. Many studies are based on relatively small patient cohorts, frequently involve single-center populations, and often use cross-sectional rather than longitudinal designs. In addition, substantial heterogeneity exists regarding disease stage, treatment status, biological specimens analyzed, and laboratory methodologies employed for MDA quantification.

This variability complicates direct comparisons among studies and may partly explain differences in reported MDA concentrations. Furthermore, although elevated MDA levels are consistently associated with oxidative stress, MDA is not specific to malignant disorders and may be influenced by age, diet, smoking habits, inflammatory conditions, metabolic disorders, cardiovascular diseases, and other comorbidities. Consequently, the currently available data support an association between MDA and disease-related oxidative injury, but are insufficient to establish standardized diagnostic, prognostic, or predictive thresholds for routine clinical use. Prospective multicenter studies using standardized analytical methodologies will be necessary to better define the clinical utility of MDA across different hematological malignancies.

An important distinction should be drawn between clinical observations and mechanistic evidence generated in experimental systems. While elevated MDA levels have been consistently documented in patients with various hematological malignancies, many of the proposed therapeutic implications of MDA, particularly those related to ferroptosis induction and redox-targeted interventions, arise primarily from in vitro and preclinical investigations.

Therefore, current evidence supports MDA as a biomarker reflecting oxidative stress and lipid peroxidation, whereas its role as a clinically useful diagnostic, prognostic, predictive, or pharmacodynamic marker remains to be established through prospective multicenter clinical studies.

The findings presented in this review should also be interpreted in light of the limitations inherent to its narrative design. Although the literature search was structured and included major biomedical databases, the review was not conducted according to a predefined systematic review protocol, and no formal quality assessment or quantitative synthesis of the included studies was performed. Consequently, study selection and evidence interpretation may have been influenced by the characteristics of narrative synthesis. Future systematic reviews and meta-analyses using standardized methodological frameworks will be necessary to provide more robust estimates of the clinical and biological significance of MDA across different hematological malignancies.

Overall, MDA represents a promising but context-dependent biomarker at the intersection of OS, LPO, and hematologic malignancy biology. Future studies should aim to standardize analytical methods, define clinically meaningful cut-off values, evaluate longitudinal changes during treatment, and integrate MDA assessment into broader redox and molecular profiling approaches. Such efforts may clarify whether MDA can be used not only as a marker of oxidative damage, but also as a tool for monitoring disease activity, identifying redox vulnerabilities, predicting treatment-related toxicity, and evaluating therapies targeting LPO and ferroptosis in hematologic malignancies.

## Figures and Tables

**Table 1:** Biological and clinical significance of malondialdehyde (MDA) across major hematological malignancies.

Hematological Malignancy	Representative Quantitative Findings on MDA	Main Biological Associations	Potential Clinical Implications	Key References
Acute Myeloid Leukemia (AML)	MDA levels approximately 7-fold higher than in healthy controls; persistently elevated at relapse	ROS overproduction, antioxidant depletion, DNA damage, ferroptosis susceptibility	Disease monitoring, relapse prediction, assessment of ferroptosis-targeted therapies	Rasool et al. [24]; Zhou et al. [21]
Chronic Myeloid Leukemia (CML)	Significantly higher MDA levels than controls; further increase in imatinib-resistant patients	BCR::ABL1-driven ROS generation, lipid peroxidation, clonal evolution	Evaluation of therapeutic resistance and disease progression	Ahmad et al. [35,36]; Ammar et al. [37]
Polycythemia Vera (PV)	Approximately 6-fold increase in serum MDA compared with healthy individuals	Erythrocyte membrane damage, endothelial dysfunction, oxidative vascular injury	Stratification of thrombotic risk and treatment monitoring	Durmus et al. [38]
Essential Thrombocythemia (ET)	Significantly elevated MDA levels; reduced after cytoreductive treatment	Platelet activation, chronic inflammation, oxidative imbalance	Identification of patients at increased vascular risk	Durmus et al. [39]; Musolino et al. [40]
Primary Myelofibrosis (PMF)	Circulating MDA concentrations approximately 5-fold higher than controls	Chronic inflammation, fibrosis, antioxidant depletion, metabolic dysfunction	Evaluation of disease burden and progression	Mangione et al. [41]; Vener et al. [42]
Hodgkin Lymphoma (HL)	Significantly increased serum MDA associated with reduced total antioxidant capacity	Tumor-associated inflammation, mitochondrial dysfunction, redox remodeling	Biomarker of disease activity and long-term treatment sequelae	Morabito et al. [43]; Marini et al. [44]
Aggressive Non-Hodgkin Lymphoma (NHL)	Elevated MDA associated with increased protein carbonylation and reduced antioxidant defenses	Oxidative DNA damage, inflammation, immune dysregulation	Prognostic biomarker and indicator of oxidative burden	Haddouche et al. [45]; Zahzeh et al. [46]
Multiple Myeloma (MM)	Increased MDA detectable from stage I disease and progressively rising with disease stage	Endoplasmic reticulum stress, bone disease, inflammation, lipid peroxidation	Disease monitoring, assessment of bone involvement and treatment toxicity	Smirnova et al. [47]; Khadem-Ansari et al. [48]; Gangemi et al. [49]

Elevated MDA levels consistently reflect increased lipid peroxidation and redox imbalance, with potential diagnostic, prognostic, and monitoring application. Whenever available, quantitative estimates of MDA elevation relative to healthy controls were included to facilitate comparison across different hematological malignancies.

**Table 2:** Emerging role of malondialdehyde (MDA) as a biomarker of ferroptosis and redox-targeted therapeutic strategies in hematological malignancies. MDA may serve as a pharmacodynamic indicator of lipid peroxidation-induced cell death and treatment response.

Hematological Malignancy	Experimental/Therapeutic Context	MDA Findings	Key Molecular Targets	Potential Clinical Relevance	References
AML	GPX4 inhibition	Significant increase in lipid peroxidation and MDA generation during ferroptosis induction	GPX4	Monitoring of ferroptosis-targeted approaches	Akiyama et al. [31]
AML	Cytarabine-resistant AML cells with SIRT1 inhibition	Increased intracellular MDA accompanied by ferroptosis restoration	SIRT1, HMGB1, ACSL4	Overcoming drug resistance	Kong et al. [33]
AML	MLN4924 (pevonedistat) treatment	Marked MDA accumulation reversed by ferrostatin-1	SLC7A11, GPX4	Pharmacodynamic biomarker of ferroptosis	Jian et al. [34]
AML	Hypomethylating agents combined with ferroptosis inducers	Increased MDA secondary to enhanced lipid peroxidation	MAGEA6, AMPK, SLC7A11, GPX4	Combination treatment strategies	Feng et al. [30]
CML	TKI-resistant experimental models	MDA accumulation associated with enhanced ferroptosis susceptibility	GPX4, ENO1, glutathione pathway	Potential strategy to overcome TKI resistance	Peng et al. [55]
NHL	Artesunate plus sorafenib treatment	Significant increase in MDA during ferroptosis-mediated lymphoma cell death	STAT3, GPX4	Biomarker of treatment response	Chen et al. [72]
MM	Emerging ferroptosis-related studies	MDA reflects iron-driven lipid oxidative damage and redox imbalance	Iron-lipid peroxidation axis, antioxidant systems	Development of redox-modulating therapies	Nasso et al. [82]; Allegra et al. [85]
Ph-negative MPNs	Chronic inflammation and oxidative stress-driven disease evolution	Persistently elevated MDA associated with disease activity and thrombosis risk	JAK-STAT signaling, inflammatory pathways	Monitoring of disease burden and response to antioxidant strategies	Durmus et al. [38,39]; Mangione et al. [41]

**Legend:** MDA = malondialdehyde; GPX4 = glutathione peroxidase 4; AML = acute myeloid leukemia; CML = chronic myeloid leukemia; NHL = non-Hodgkin lymphoma; MM = multiple myeloma; MPNs = myeloproliferative neoplasms; ACSL4 = Acyl-CoA Synthetase Long-Chain Family Member 4; MAGEA6 = Melanoma Antigen Family A6; AMPK = AMP-Activated Protein Kinase; JAK/STAT = Janus Kinase/Signal Transducer and Activator of Transcription signaling pathway; ENO1 = Alpha-Enolase (Enolase 1).

## Data Availability

No new data were created or analyzed in this study. Data sharing is not applicable to this article.

## Abbreviations

The following abbreviations are used in this manuscript:

AGEs	Advanced Glycation End Products
AML	Acute Myeloid Leukemia
AOPP	Advanced Oxidation Protein Products
BCR::ABL1	Breakpoint Cluster Region-Abelson Fusion Gene
CML	Chronic Myeloid Leukemia
DNA	Deoxyribonucleic Acid
ET	Essential Thrombocythemia
Fe^2+^	Ferrous Iron
GPX4	Glutathione Peroxidase 4
GSH	Reduced Glutathione
HL	Hodgkin Lymphoma
H6PD	Hexose-6-Phosphate Dehydrogenase
HMGB1	High-Mobility Group Box 1
LPO	Lipid Peroxidation
MDA	Malondialdehyde
MM	Multiple Myeloma
MPNs	Myeloproliferative Neoplasms
NADPH	Nicotinamide Adenine Dinucleotide Phosphate
NHL	Non-Hodgkin Lymphoma
ORAC	Oxygen Radical Absorbance Capacity
OS	Oxidative Stress
OSI	Oxidative Stress Index
PMF	Primary Myelofibrosis
PUFAs	Polyunsaturated Fatty Acids
PV	Polycythemia Vera
ROS	Reactive Oxygen Species
SIRT1	Sirtuin 1
SLC7A11	Solute Carrier Family 7 Member 11
SOD	Superoxide Dismutase
STAT3	Signal Transducer and Activator of Transcription 3
TAC	Total Antioxidant Capacity
TAS	Total Antioxidant Status
TKI	Tyrosine Kinase Inhibitor
TOS	Total Oxidative Status
TRX	Thioredoxin
8-OHdG	8-Hydroxy-2′-Deoxyguanosine
